# Generative vocal plasticity in chimpanzees

**DOI:** 10.1016/j.isci.2025.112381

**Published:** 2025-04-08

**Authors:** Adriano R. Lameira, Bruno Caneco, Arik Kershenbaum, Guillermo Santamaría-Bonfil, Josep Call

**Affiliations:** 1 ApeTank, Department of Psychology, University of Warwick, Coventry, UK; 2 DMP Statistical Solutions, St Andrews, UK; 3 Department of Zoology, University of Cambridge, Cambridge, UK; 4 Girton College, University of Cambridge, Cambridge, UK; 5 Data Portfolio Manager Department, BBVA Mexico, Mexico City, Mexico; 6 Department of Psychology and Neuroscience, University of St Andrews, St Andrews, UK

**Keywords:** Wildlife behavior, Zoology, Evolutionary biology

## Abstract

Modern theory posits that human-ape differences in voice command account for speech evolution. However, comparison has been indirect and conjectural based on vocal learning taxa far related from Hominids, instead of direct and quantitative based on great ape calls that, like all speech sounds, are local-specific and non-universal to the species. Moreover, the null hypothesis that the great ape voice command is purely reflexive has never been directly tested. Here, we show that in controlled, constant experimental settings, captive chimpanzees exhibit high-dimensional dexterity over voice activation and modulation in two atypical vowel-like calls. Subjects made unrestricted, multidimensional, and distinct voice changes within and between individuals, inducing parameter changes up to 10,000%, rejecting null hypothesis’ predictions. Forecasting models indicated unmitigated voice novelty, altogether demonstrating emancipated and vast real-time voice control. Findings show that, contrary to traditional assumptions, speech and song evolution likely hinged on prolific voice command already available in ancestral ape-like ancestors.

## Highlights

• Controlled experiments induced atypical vowel-like calls in captive chimpanzees
• Chimpanzees exhibited multidimensional, emancipated, and regenerative voice control
• Findings refute the common hypothesis of reflexive vocal production in great apes
• Ape-like ancestors had pre-existing capacities for speech and song evolution

## Graphical abstract

**Figure:**
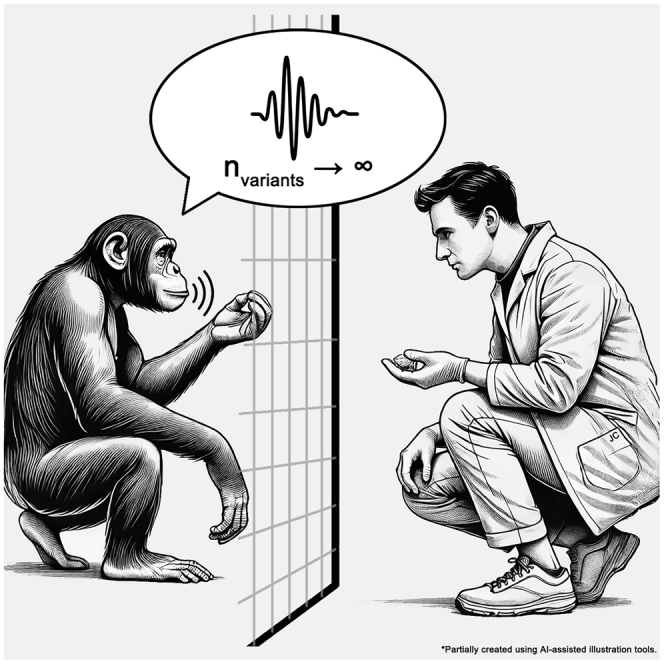

## Introduction

Nonhuman great apes can produce both voiced vowel-like calls^1^^,^^2^ and voiceless consonant-like calls.^3^^,^^4^^,^^5^^,^^6^ If archetypes for the two fundamental building blocks of all the worlds’ spoken languages are available in our closest living relatives, why and how has speech evolved only in humans?

According to the “Kuypers and Jürgens” or neural hypothesis,^7^^,^^8^^,^^9^ great apes lack the neural circuitry that endows motor control over vocal production learning, seemingly rendering great apes unable to control voice activation and modulation and, thus, expand their repertoire with new intonations or voiced call variants.^10^^,^^11^^,^^12^^,^^13^ Counterintuitively, Jürgens never studied great apes and Kuypers described the neural circuitry in the chimpanzee brain that is allegedly absent according to the Kuypers & Jürgens hypothesis.^14^^,^^15^ Analogous circuitry and vocal learning capacities have convergently evolved across distinctly different brains and bodies in some bird and mammal groups (e.g., songbirds,^16^^,^^17^ seals,^18^ parrots,^19^ whales,^20^^,^^21^ elephants,^22^^,^^23^ bats,^24^ hummingbirds,^25^ and walruses^26^^,^^27^); however, these cases can neither shed light over speech precursors in ancient hominids nor explain why there are no linguistic birds, elephants, or walruses.

Vocal learning has shown predictive empirical power within one clade, namely birds,^28^ but extrapolating across far-related taxa has proven inaccurate, unsystematic, and ambiguous.^14^^,^^29^ For example, vocal learning in belugas is accomplished via control of the vestibular air sac,^20^ in elephants via control of the trunk,^30^ and in birds via control of the syrinx, a vocal organ that can contain up to three sound sources,^31^ each one functionally analogous to a set of vocal folds in the mammalian larynx.^32^ Describing vocal learning with binary (vocal learners vs. non-vocal learners)^7^^,^^10^ or unidimensional terms (weak > intermediate ≫ advanced vocal learners),^33^ as used to date,^7^^,^^11^^,^^12^ does not provide the necessary detail for objectively capturing these differences and how distinct brains came to control distinct bodies and sound sources.^14^^,^^29^ Vocal learning as a qualitative benchmark of vocal capacities across taxa is no longer tenable. A measurable and quantitative approach is warranted to avoid skewed comparisons of what species can and cannot do vocally,^14^^,^^29^ notably within the Hominid family.

Among great apes, above and beyond the most recent shared ancestry with humans, not all vocal behaviors provide equal comparative validity or evolutionary insight. For example, cries and screams are found in similar form and function in all primates, including humans.^34^ Speech evolved without replacing these vocalizations. Cries and screams were, hence, improbable targets of the selective pressures that drove human vocal control and speech. Drawing a biologically valid human-ape comparison requires specific vocal behaviors to avoid comparing proverbial apples with oranges.

Speech is universal to the human species, but *no* speech sound (i.e., no vowel or consonant) is universal across the world’s languages^35^^,^^36^ — speech sounds are local- and individual-specific.^37^^,^^38^^,^^39^ Accurate human-ape comparisons must, thus, be based on “atypical” great ape calls^5^^,^^40^^,^^41^^,^^42^^,^^43^ that likewise reflect local- and individual-specific histories and social contexts.^5^^,^^42^^,^^43^^,^^44^^,^^45^^,^^46^^,^^47^^,^^48^^,^^49^^,^^50^^,^^51^ Similarly to any speech sound, atypical great ape calls exhibit geographic patterns that are best explained by local modification and innovation followed by social learning and cultural transmission.^5^^,^^42^^,^^43^^,^^44^^,^^45^^,^^46^^,^^47^^,^^48^^,^^49^^,^^50^ They can take the form of voiceless consonant-like^5^^,^^41^^,^^43^^,^^45^^,^^46^^,^^48^^,^^49^^,^^52^ and voiced vowel-like calls,^41^^,^^42^^,^^43^^,^^44^^,^^45^^,^^48^^,^^49^ which can be produced at speech-like rhythm in direct articulatory and acoustic homology with humans.^43^^,^^53^ In the wild, these combinations have been shown to be governed by advanced control skills^6^ and combinatorial rules,^54^^,^^55^^,^^56^^,^^57^ used as canvas for high-order^58^^,^^59^ and language-like cognitive capacities, such as communication about past events^60^ and deception.^61^^,^^62^ Curiously, bird and whale song, often heralded as *the* best examples of vocal learning in nonhuman animals,^63^ are composed by song elements that often do not reach universality in the species^17^^,^^64^^,^^65^ in a clear parallel with great ape atypical calls.

Great ape atypical calls produced in captivity toward humans are of special empirical value. Captivity allows for a controlled setting and a level of context stability that cancels most extraneous effects that could bear weight on voice production changes (e.g., body position and movement,^66^^,^^67^^,^^68^ arousal and audience effects,^69^^,^^70^^,^^71^^,^^72^^,^^73^^,^^74^ contextual variation,^4^ variation in surrounding acoustics).

Here, to reliably measure and quantify laryngeal control and voice production in great apes, we analyzed two atypical, voiced calls used by two captive chimpanzees at the Leipzig Zoo, Germany (Riet, Studbook ID: 11709; Alex, 13116), which the two individuals used to gather the attention of, and request food from, human caretakers. In two experimental sessions with each subject, a familiar human experimenter (J.C.) provided familiar food items to elicit the target idiosyncratic vocalizations from each subject while he or she was alone in its indoor quarters, in a total of four sessions (i.e., two sessions for each subject). Experimental conditions were always kept constant during and across sessions, such that there were no changes in food type, human-to-ape relative position and distance, recording equipment, and surrounding acoustics.

The number of eligible great ape individuals in captivity was critically limited by those who are known to produce atypical vocalizations, produce these vocalizations in non-undesirable conditions (e.g., hard stereotypies), and live in zoos supportive and accommodating of research. These conditions made sample sizes necessarily small. Accordingly, the study was set in a clear hypothesis testing form. Following Black Swan’s razor,^75^ one deviant individual is sufficient to falsify an hypothesis that claims universality and/or uniformity across a population or species. As such, if inconsistent with the hypothesis under test, results from 1, 2 or 10, 20, 100 or 200 individuals provide the same heuristic value for rejecting that hypothesis.

**Figure 1:**
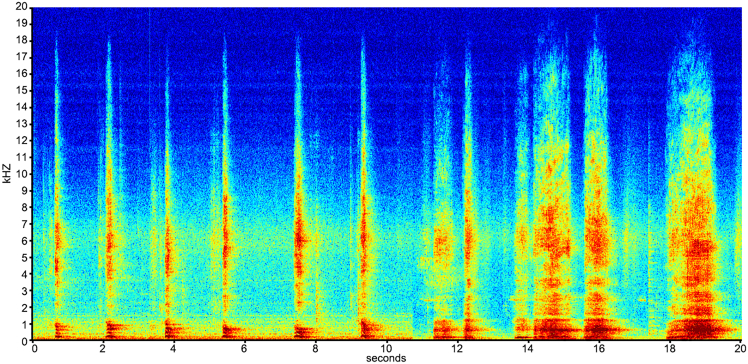
Spectrographic representation of the two chimpanzee atypical vowel-like calls Six calls produced by Alex, followed by 6 calls produced by Riet. Spacing between calls has been altered for visual clarity (see Audio S1).

We extracted four acoustic parameters from call recordings using Raven Pro (Version 1.6.3, Cornell Lab); two parameters characterizing “voice activation” [maximum frequency (“maxfreq”; Hz) and duration (s), which measure the dominant and loudest frequency at which the laryngeal source oscillates (predominantly, the vocal folds) and for how long, respectively], and two parameters characterizing “voice modulation” [peak frequency contour average slope (“freqslope”; Hz) and average acoustic entropy (bits), which measure the average change in oscillation rate of the source and how regular/chaotic that oscillation is,^76^ respectively] (Figure 1; Audio S1). On the basis of these parameters, we used three levels of analysis to test directly the traditional Kuypers and Jürgens hypothesis^7^^,^^8^^,^^9^ that poses that great apes lack laryngeal control, and consequently, that voice production is purely hardwired, automatic, and reflexive. We analyzed voice activation and modulation (1) across subjects, (2) between subjects, and (3) between sessions within subjects. If the traditional hypothesis is correct, voice activation and modulation should be stereotypical and constrained in (1) and equal in level and range in (2) and (3).

Audio S1. Exemplary recordings of six calls produced by Alex, followed by 6 calls produced by Riet

## Results

### Directional and distance change in acoustic space

**Figure 2:**
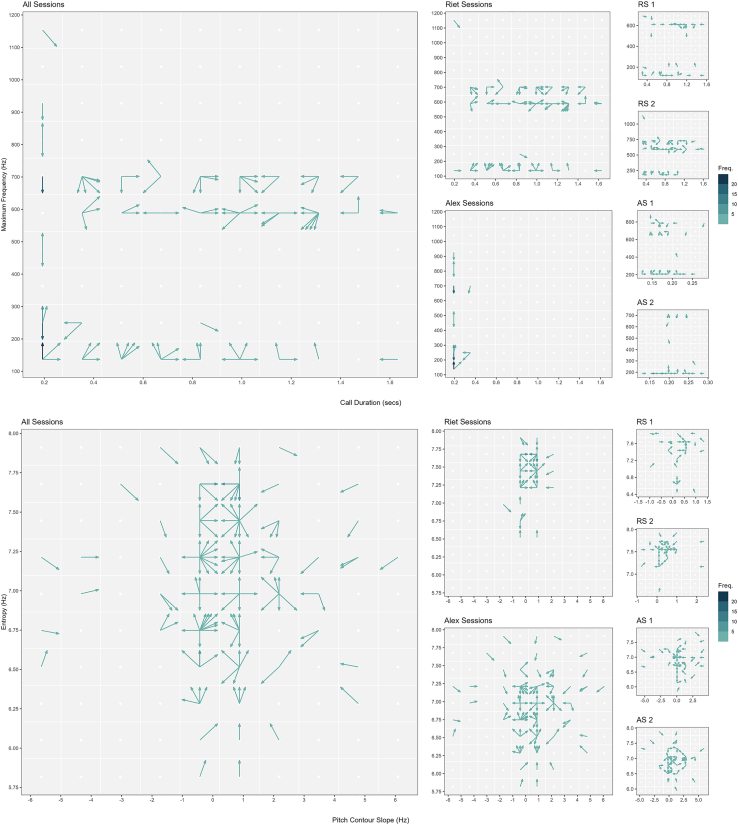
Directional change of atypical chimpanzee vowel-like voiced calls in acoustic space This figure depicts (with arrows) the possible direction change(s) that a call’s features made after occupying a particular cell within the acoustic space. Upper panel (maximum frequency x duration) depicts direction changes in voice activation, lower panel (entropy x pitch contour slope) depicts direction changes in voice modulation. Columns depict voice activation and modulation (*i*) across subjects, (*ii*) between subjects and (*iii*) between sessions within subjects. Colour saturation depicts frequency (i.e., rate) of calls within a cell making the same direction change in acoustic space.

Figure 2 depicts chimpanzee atypical, vowel-like voiced calls in acoustic space and the direction change taken from each acoustic position to a subsequent one for (1), (2), and (3). Each voice change entailed idiosyncratic voice action. Some positions in the acoustic space showed no voice occurrence (i.e., subjects did not vocalize in that maxfreq/duration or freqslope/entropy combination), some positions exhibited only one change direction (i.e., voice change in maxfreq *or* duration and freqslope *or* entropy as horizontal or vertical movement in the acoustic space), whereas other positions presented multiple change directions (i.e., voice change in maxfreq *and* duration and freqslope *and* entropy as diagonal movement in the acoustic space). Across subjects (1), voice activation exhibited a range of >1000Hz in maxfreq and >1.5s in duration, and voice modulation a range of ∼12Hz in freqslope and >2bit in entropy. This rejected the prediction that great ape voice action in constant context is stereotypical and constrained. Indeed, these were unusually large ranges, especially for voice activation. Had these values been observed in the wild, they would licence the creation of multiple call types and would, according to signal theory, be assumed to be produced across distinct contexts or populations.^4^ Between subjects (2), voice activation and modulation were distinct, with each individual navigating voice action in different regions and differently across the acoustic space. This was also the case to a lesser degree between sessions (3). This rejected the prediction that voice action ought to be equal in level and range across subjects and sessions.

**Figure 3:**
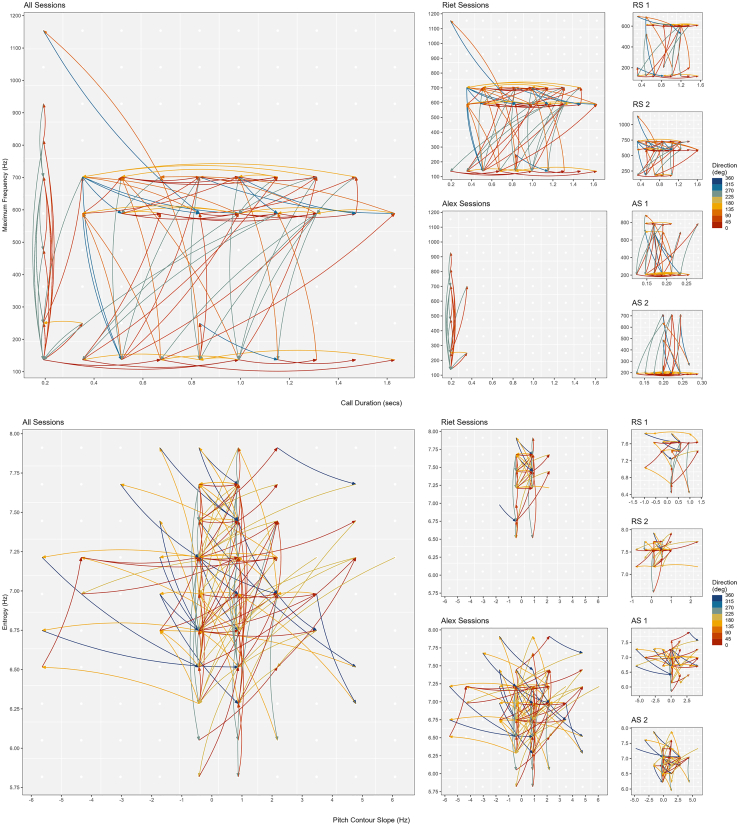
Distance change of atypical chimpanzee vowel-like voiced calls in acoustic space This figure complements Figure 2 and depicts (with arrows) the distance of change(s) that a call’s features made after occupying a particular cell within the acoustic space. Upper panel (maximum frequency x duration) depicts voice activation, lower panel (entropy x pitch contour slope) depicts voice modulation. Columns depict voice activation and modulation (*i*) across subjects, (*ii*) between subjects and (*iii*) between sessions within subjects. Colour depicts direction of movement (in angle degrees).

Figure 3 provides a similar graphic representation but depicting and highlighting the distance of voice changes taken from each major position in the acoustic space, showing that individuals engaged in free exploration of the acoustic space. Subjects performed voice changes in multiple directions *in combination with* multiple possible distances *and* at different moments of their vocal exchange with the human experimenter.

### Absolute and percentual change in acoustic space

**Figure 4:**
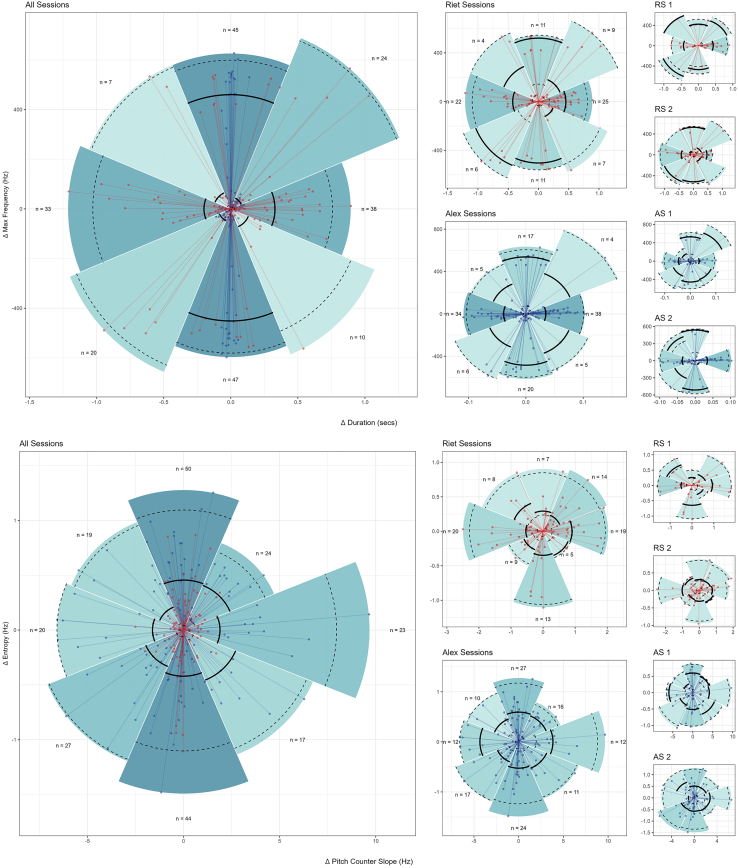
Absolute change of atypical chimpanzee vowel-like voiced calls in acoustic space Upper panel (maximum frequency x duration) depicts voice activation, lower panel (entropy x pitch contour slope) depicts voice modulation. Columns depict voice activation and modulation (*i*) across subjects, (*ii*) between subjects and (*iii*) between sessions within subjects. Colour saturation depicts number of calls changing in the same major direction. Solid lines mark the median distance of points from origin within each slice, while dashed lines denote the 2.5% and 97.5% percentiles of these distances.

To obtain an enhanced representation of the degree and range of voice change used by chimpanzees, we plotted voice activation and modulation in terms of *relative* voice change (Figure 4), where the origin point of the graph represents a call variant state connected to the variant that followed as a colored point. For (1), (2), and (3), voice change was radial and multidimensional, that is, subjects experienced virtually no impediment against voice change. For voice activation, this meant that subjects were able to: First, move in duration negatively or positively while maintaining constant maxfreq (i.e., change along the horizontal axis of the acoustic space); second, move in maxfreq negatively or positively while maintaining constant duration (i.e., change along the vertical axis); third, move in both duration and maxfreq negatively or positively (i.e., lower-left and upper-right quadrants); and, fourth, move in duration and maxfreq in opposite directions (i.e., upper left and lower right quadrants).

The same pattern of free voice action was observed with regard to voice modulation. “Pizza slice” size and range lines (i.e., bold and dashed lines) for (1) and (2) show that there was no uniform, constant, or confined disposition for voice activation or modulation, while (3) confirmed that voice change was dynamic and unregimented between sessions for the same individual. The assumption that across (1), (2), and (3), voice change ought to be small and/or portray unidirectional and/or stereotypical voice changes, as predicted by the traditional Kuypers and Jürgens hypothesis, received no empirical support.

**Figure 5:**
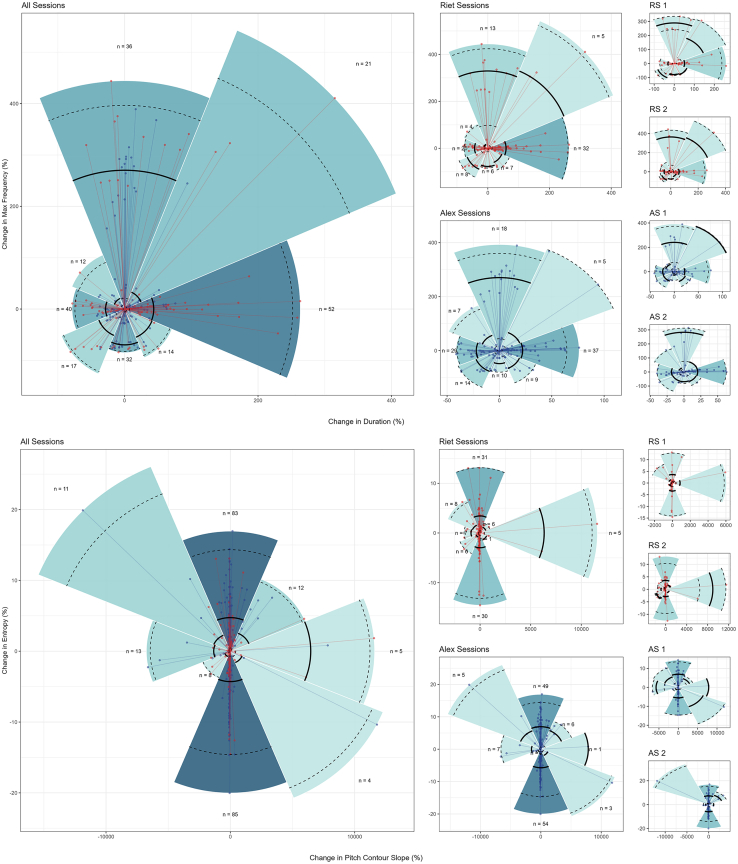
Percentual change of atypical chimpanzee vowel-like voiced calls in acoustic space Upper panel (maximum frequency x duration) depicts voice activation, lower panel (entropy x pitch contour slope) depicts voice modulation. Columns depict voice activation and modulation (*i*) across subjects, (*ii*) between subjects and (*iii*) between sessions within subjects. Colour saturation depicts number of calls changing in the same major direction. Solid lines mark the median distance of points from origin within each slice, while dashed lines denote the 2.5% and 97.5% percentiles of these distances.

Figure 5 provides a similar graphic representation plotted in terms of percentual voice change. Multiple voice changes in activation and modulation surpassed 100% and, on several occasions, went beyond a 400% and 10,000% change in voice parameters for both individuals, respectively. This demonstrates subjects were not producing simple, subtle, or subperceptual sub-variants. Subjects exercised voice control to an extent that was unambiguous and that would typically be interpreted as encoding biologically distinct contexts or meanings, both by conspecifics^77^ and human observers.^69^ Overall, percentual voice change corroborated the view that individuals volitionally engaged in real-time voice action between each other and between sessions. These differences were statistically confirmed, with subjects using significantly different sequence transitions between voice activation and modulation states [permutation-based test for normalized mutual information (NMI): voice activation: NMI scores, (A) 0.101, (B) 0.869, *p* < 0.001; voice modulation: (A) 0.543, (B) 0.769, *p* < 0.001].

### Emergence of new states in acoustic space

To directly test the generative voice capacity of the chimpanzee subjects, we calculated per acoustic parameter, the moving Shannon entropic emergence,^47^^,^^78^^,^^79^ which describes the degree to which new states appear in a system across time, where emergence 0 describes a system where the same state repeats incessantly and where emergence 1 describes a system exhibiting ever-changing states. According to the traditional hypothesis, great ape vocal control between individuals and sessions should exhibit emergence ∼0, corresponding to stereotypical, unyielding, and constrained vocal command.

**Figure 6:**
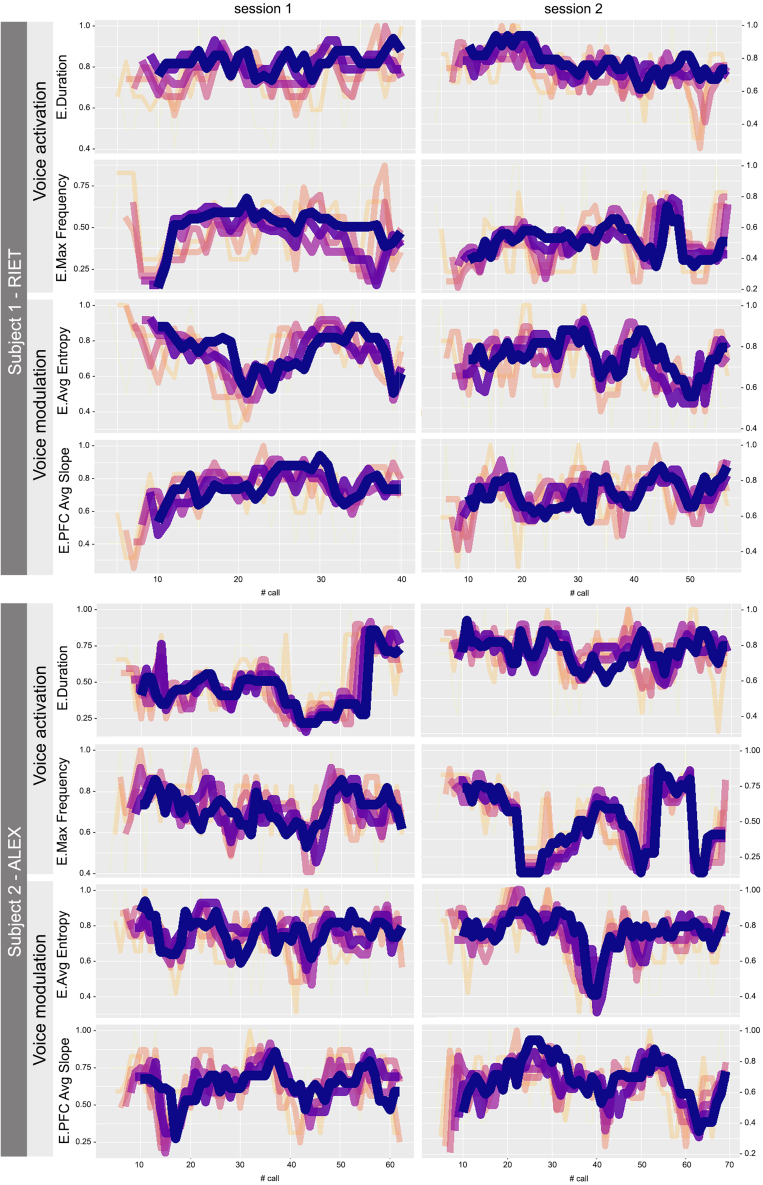
Moving entropic emergence of atypical chimpanzee vowel-like voiced calls per session per individual Colours depict 5 (light orange) to 10-step (dark blue) moving emergence.

Figure 6 represents the moving emergence over time per individual per session. The rate of appearance of new parameters variants was ever-changing, denoting meta-variation in chimpanzee’s voice command – that is, *voice variation varied* across time. Each parameter’s moving emergence briefly approached 0.2 on occasions but was overall above 0.5 across all sessions, indicating a constant current of new voice states entering the sessions. Within the same session, each parameter’s emergence was unrelated with that of the others. For example, for voice activation, high and low emergence of duration variants did not correspondingly coincide with instances of high or low emergence of max frequency states. The same was observed for voice modulation parameters. This demonstrates different efferent neuro-motoric control mechanisms. Between sessions and individuals, the two subjects exhibited different emergence regimes, once again representative of off-the-cuff voice control and production. Overall, results empirically refute the traditional theory and confirm that there are various independent voice command mechanisms and neuro-motoric circuitry at work during voiced call production in great apes.

To verify whether the two individuals would have exhausted their supply of new voiced variants shortly after the end of our experimental sessions, we conducted an ARFIMA forecasting model for repertoire emergence. The expectation was that emergence would drift or drop to 0 in the moment individuals had deployed all their possible voice variants, that is, exhausted their voice activation and modulation range. Had our experimental sessions continued for an extra of 50% of the time, no emergence level would have dropped to zero. For both voice activation and voice modulation, Riet and Alex exhibited asymptotic emergence trend levels, with average values around 0.5 or above for both individuals across sessions (Figure S1), that is, both individuals in both sessions would have theoretically continued to generate new voice variants at generous rates into the future. In other words, we would have heard new voice activation and modulation combinations that had not yet been heard so far, denoting an unexhausted voiced repertoire.

## Discussion

Our findings demonstrate that chimpanzees have emancipated control over their larynxes and production of voiced vowel-like atypical calls, giving them vast manoeuvrability across sound space. Subjects continuously produced new voice states during the span of the experimental sessions and their repertoire showed no signs of depletion of new voice states into the future. Voice command was not accountable by automatic, arousal or other reflex-based mechanism, as argued by the Kuypers and Jürgens hypothesis.^7^^,^^8^^,^^9^ For example, our observations would have been impossible had the subjects relied on conditioning learning. Chimpanzees would have been predicted to converge voice production toward a variant that they presumed to be associated with, or best predict, food delivery. This was not the case. Arousal and motivation can affect parameters of vocal behavior, and they cannot be subsided non-invasively in animals voluntarily participating in a free interaction with a human. Nonetheless, arousal and motivation cannot be invoked as sufficient and exclusive explanation of our results for three reasons. First, there were no gradual changes along vocal parameters during experimental sessions to suggest oscillations in affective physiology. Second, the sheer number of vocal changes observed across various axes in the acoustic space would imply that, in the course of a few minutes, individuals experienced thousands of distinct homeostatic, affective, or arousal states. Third, our entropy analyses show that there were no gradual changes in the production rate of new voice variables, disproving low-level factors or mechanisms.

Unlike past, grand sweaping statements about great ape vocal command, our findings derive from measurable, quantitative parameters in a direct hypothesis-testing approach. Findings are consistent with evidence from other chimpanzee vocal behavior in captivity^41^^,^^48^^,^^49^^,^^50^^,^^80^ and the wild,^69^^,^^77^ as well as their neurology^14^^,^^81^^,^^82^^,^^83^ and the vocal behavior of other nonhuman great apes.^6^^,^^40^^,^^42^^,^^43^^,^^44^^,^^45^

This level of voice control in chimpanzees raises the intriguing possibility of call cultures in captivity across zoos. Our findings demonstrate that the existence and extent of these cultures should not be dismissed or underestimated before comprehensive effort is dedicated to directly chart vocal repertoires across zoos (“absence of evidence is not evidence of absence”). Multiple instances of vocal innovation in all great ape genera have been described^5^^,^^40^^,^^41^^,^^42^^,^^43^^,^^44^^,^^48^^,^^49^^,^^50^^,^^52^^,^^80^ and remain overall under-reported. Instances of ape-to-ape^5^^,^^48^ and human-to-ape vocal learning^43^^,^^80^ show that there are conditions and factors that foster social learning and cultural dissemination of novel calls. Science remains largely oblivious about these conditions and factors, however, there is renewed understanding of why earlier historical efforts to teach great apes to speak failed.^84^^,^^85^ Avoiding ethical issues incurred by classic great ape language projects will bring clarity about how humans can engage with captive great apes to help enrich their communicative lives.

Our findings upend decades-old claims that great ape voice control is qualitatively distinct from humans’. Speech and song evolution likely rested on prolific voice command of an ape-like hominid ancestor and its underlying neuro-motoric substrates.

## Resource availability

### Lead contact

Further information and requests for resources should be directed to the lead contact, Adriano R. Lameira (adriano.lameira@warwick.ac.uk).

### Materials availability

No new materials were generated in this study.

### Data and code availability

• Data reported in this paper are available from the lead contact upon request, subject to their approval.
• All original code has been deposited in GitHub and will be made publicly available as of the date of publication. Link is listed in the key resources table.
• Any additional information required to reanalyse the data reported in this paper is available from the lead contact upon request, subject to their approval.

## Acknowledgments

A.R.L. was supported by the 10.13039/100014013UK Research and Innovation, Future Leaders Fellowship grant agreement number MR/T04229X/1.

## Author contributions

Conceptualization: A.R.L. Methodology: A.R.L. and J.C. Formal analysis: A.K. and G.S.-B. Visualization: A.R.L. and B.C. Writing original draft: A.R.L.. Writing–review and editing: A.R.L., B.C., A.K., G.S.-B., and J.C.

## Declaration of interests

Authors declare no conflict of interests. G.S.-B. clarifies that the opinions and research presented in this article are his own and do not necessarily reflect those of BBVA México.

## STAR★Methods

### Key resources table

**Table:** 

REAGENT or RESOURCE	SOURCE	IDENTIFIER
**Deposited data**		

Repository data and R scripts	This paper	https://github.com/bcaneco/chimpanzee_calls_plots

**Experimental models: Organisms/strains**		

Chimpanzee (*Pan troglodytes verus)*	Wolfgang Köhler Primate Research Center, Leipzig Zoo, Pfaffendorfer Str. 29, 04105 Leipzig, Germany: Riet (Studbook ID: 11709; female born in 1977; 40yo at time of study) and Alex (13116; male born in 2001; 16yo at time of study).	https://www.zoo-leipzig.de/en/

**Software and algorithms**		

R v4.2.1	R Core Team. R: A Language and Environment for Statistical Computing. R Foundation for Statistical Computing^86^	www.r-project.org; RRID:SCR_001905

### Experimental model and study participant details

Audio recordings were collected at the Wolfgang Köhler Primate Research Center, Leipzig Zoo, Germany, during the summer of 2017. Our two chimpanzee subjects were Riet (Studbook ID: 11709; female born in 1977; 40yo at time of study) and Alex (13116; male born in 2001; 16yo at time of study). Even though the two subjects live in separate groups, they had been within mutual earshot on a daily basis and have lived at this facility for more than 15 years.

#### Ethics approval

This study was approved by the University of St Andrews’ Animal Welfare and Ethics Committee (AWEC), and a joint ethical committee of the Max Planck Institute for Evolutionary Anthropology and Leipzig Zoo.

### Method details

#### Data collection

We used the built-in microphones of a H4n Zoom Handy recorder (ZOOM Corporation, Tokyo, Japan) at ∼0.5m away through enclosed mesh that separated the subjects and a familiar human experimenter at all times. During recordings, the human experimenter (JC) showed, momentarily withheld, and provided familiar food items to elicit vocalizations as food-requests from the subjects. Experimental conditions were kept constant at all times during the sessions, assuring there were no changes in food type rewards, position of human experimenter relative to the subject or along the enclosure space. We recorded these human-directed vocalizations from two unrelated chimpanzee individuals, Riet and Alex. Two recording sessions were collected from each individual while they were alone in their indoor quarters. The atypical calls produced by both subjects were strictly known to be produced in this context: to gather the attention of human to request food. This context-specificity was determined through 18 years of continuous behaviour of observation.

### Quantification and statistical analysis

#### Data measures

Recordings were transferred to a computer with a sampling rate of 44.1 kHz. For acoustic comparison, we used Raven interactive sound analysis software (version 1.5, Cornell Lab of Ornithology, Ithaca, New York; window type: Hann; 3-dB filter bandwidth: 124 Hz; grid frequency resolution: 2.69 Hz; grid time resolution: 256 samples) to manually extract four acoustic parameters from the subjects’ vocalizations: max frequency (Hz; i.e. that of highest dB/loudest; a close proxy of pitch^5^), duration (seconds), average entropy (bits) and average pitch contour slope (Hz). Parameters were extracted by manually drawing a selection box in the spectrogram view encompassing the length/range of each vocalization along the time/spectral axes.

#### Data rendering

Directional (Figure 2), distance (Figure 3), absolute (Figure 4) and percentual change graphs (Figure 5) were built with tailor made scripts (available in https://github.com/bcaneco/orangutan_calls_plots) using R and the following R packages: colortools v0.1.5,^87^ glue v1.6.2,^88^ grid v4.2.1,^86^ hexbin v1.28.2,^89^ MetBrewer v0.2.0,^90^ patchwork v1.1.2,^91^ rcartocolor v2.0.0,^92^ scales v1.2.1,^93^ sf v1.0.8,^94^ sfheaders v0.4.0,^95^ tidyverse v1.3.2.^96^

#### Sequence analyses

To statistically compare chimpanzee voice changes between individuals, we performed analyses separately for voice activation (duration and maximum frequency) and voice modulation (average entropy and average pitch contour slope). For each of case, we pooled all the calls of both subjects and performed a Gaussian mixture model clustering using the Matlab^97^ function fitgmdist on the two principal components of the parameters. We clustered using 2-20 clusters, repeating 10 times to account for random initial conditions, and calculated the mean silhouette value for the clusters, and the AIC for each additional cluster. We used both of these metrics to determine the optimal number of clusters for the mixture model. Five clusters maximised the silhouette value and was consistent with diminishing AIC variation (see supplemental information). This data treatment allowed running statistical tests that require categorical instead of continuous data, hence, dividing each call type into possible sub-categories.

Assigning each call to one of the clusters, each of which was deemed to be a call type 1-5, we represented the vocalisations as a sequence of call types and calculated the 5x5 transition tables between subjects. To calculate the transition probability between two call variants, we used a graphical representation with nodes arranged in a circular layout and arcs connecting them, where the width of each arc reflected the probability of a transition between any two call types.

To determine whether there was any statistically significant difference between the transition tables of the two subjects, we used exact tests, first measuring the similarity between transition tables with normalised mutual information NMI.^98^ We then randomly rearranged the subject/trial labels 10,000 times and recalculated the transition tables and the NMI’s between them. These NMI measurements with randomised labels formed the null distributions, against which we could interpret our true NMI similarity metric between the subjects/trials. p-values were deduced as the proportion of randomised NMI values that were more extreme (lower NMI, i.e., more different transition tables) than the true NMI value. Significance value was set at p<0.05.

#### Moving entropy analyses and forecasting models

Complexity in dynamic systems refers to the interplay of new and often unpredictable behaviors altogether with the self-organized formation of specific ones, and how, in consequence, these interactions give raise to functional/spatial/temporal structures without *a priori* specifications. Such complexity is characterized by features such as nonlinearity, emergence, self-organization, and adaptability.^99^

Shannon’s entropy measures the average amount of information or uncertainty inherent in a random variable’s possible outcomes. From it, entropy-based measures, namely Emergence (E), Self-Organization (S), and Complexity (C) can be derived (altogether ESC). The first, E, corresponds to the normalization of the entropy measured on a dynamic system, regarding its maximal entropy. Hence, 0≤E≤1, where E=1 when the system is fully random continuously producing new patterns, and E=0 when the system is fully deterministic. The second, S, measures the level of organization within the system such that 0≤S≤1. Thus, S = 1 when the system is completely ordered developing organized structures within, whereas S=0 when the system is totally at random. The third, C, is a metric of the level of interplay between E and S which allows identifying phase transitions on dynamic systems, as well as to understand the balance between order and disorder for each system.

In order to analyze dynamically the emergence, self-organization, and complexity of patterns in the time series of chimpanzees’ vocal behaviour, a univariate moving average (i.e., rolling-window) approach was used. Through calculating a moving average of ESC (*maESC*) metrics, it is possible to analyze within a time frame, the generation rate of new patterns or the stagnation in specific ones as a system evolves in time. The idea is that, by calculating these metrics, one can dynamically assess the temporal evolution of emergence, self-organization, and complexity of a dynamical system as measured by time series data.^100^ Thus, given a time series $x_{i}\in X,i=1,2,\ldots ,n$ and a window of length l<‖x‖, the *maESC* measures can be estimated as follows:

$${m_{E}}_{t}=-K\cdot \sum_{i=1}^{l}p_{t,i}\;\log_{2}p_{t,i}\text{,}$$

$${m_{S}}_{t}=1-m_{E_{t}}\text{,}$$

$$m_{C_{t}}=4*{m_{S}}_{t}*m_{E_{t}}\text{,}$$

where $m_{E_{t}},{m_{S}}_{t},\text{and}\;m_{C_{t}}$ are the moving averages of entropy-based E, S, and C of the system in time t, respectively; l is the number of different states a system has within the rolling window ending at time t, which is the same as the window size; $p_{t,i}$ is the probability of the i-th state within the window ending at time t; and K corresponds to $\frac{1}{\log\;10\left(l\right)}$. Using these definitions, the procedure for estimating the maESC is the following: i) select a window of length l, ii) for each window w and i-th state, compute the probability $p_{t,i}$, iii) calculate the $\text{ES}C_{t,i}$ values, and iv) shift the window one time step ahead and repeat from step ii). The result of this procedure are three time series ($m_{E_{t}},m_{S_{t}},m_{C_{t}}$) of length n-l.

The *maESC* approach is useful for highlighting local (i.e., within the window) changes and trends hidden by short-term fluctuations. In this sense, a consistently high moving average of emergence, $m_{E_{t}}$, indicates a system frequently producing new patterns with high variability. Conversely, a low $m_{E_{t}}$ points to a more predictable and deterministic behavior. The moving average of self-organization ($m_{S_{t}}$) highlights periods of high structural organization within the system, which may correspond to phases where the system is undergoing significant self-organization. Finally, the moving average of complexity ($m_{C_{t}}$) helps in identifying phase transitions by indicating shifts between order and disorder, thus offering a comprehensive view of the system's dynamics over time.

Here, data from the two chimpanzee individuals (ind) in two different contexts (ctx) was considered. For each ind×ctx combination, four time series corresponding to variables max frequency, duration, peak frequency contour average slope and average acoustic entropy were analyzed. For each ind×ctx×variable time series, three window sizes were initially defined, l = 4, 6, 8. It should be noted that, estimating entropy on subsequences of time series using a rolling window approach by discretization continuous observations into l states simplifies data analysis, making it computationally efficient and reducing noise.^98^ However, binning continuous data can introduce quantization errors and depends critically on the choice of binning strategy and number of bins, potentially leading to information loss.^101^ Further, the sensitivity of entropy estimates in relation to window size is also notable; smaller windows offer higher resolution but lower reliability, while larger windows provide more robust estimates at the expense of detail.^102^ Specifically, windows of size 4 and 6 estimates were noise-sensitive, in contrast, a window of size 8 offered more stable estimates, thus, l=8 is selected. These time series were used as description proxies of the temporal evolution of emergence, self-organization, and complexity of the dynamical system.

Once the *maESC* time series were estimated, it was crucial to predict the future emergence, self-organization, and complexity of chimpazees’ repertoire to estimate if individuals’ vocal repertoire would decrease or increase over time. That is, had we recorded more sessions, would we continue hearing new voice variants? Accordingly, from the resulting maESC time series obtained for each ind×ctx×variable combinations, the avg. Entropy Emergence ($m_{E_{\text{avg}.\text{Entrp}}}$) was used to carry on a forecasting procedure.

To forecast the $m_{E_{\text{avg}.\text{Entrp}}}$, first, it was required to establish an adequate forecasting model. For this purpose, several algorithms were compared to determine the best in forecasting the $m_{E_{\text{avg}.\text{Entrp}}}$ values. We compared the following forecasting algorithms: (*i*) ARIMA (AutoRegressive Integrated Moving Average), widely used for their ability to model a variety of temporal structures by combining autoregression, differencing, and moving averages^103^; (*ii*) ARFIMA (AutoRegressive Fractionally Integrated Moving Average), which extends ARIMA by incorporating fractional differencing, making it suitable for long memory processes^104^; (*iii*) ETS (Error, Trend, Seasonal), which focuses on decomposing a time series into error, trend, and seasonal components, and are particularly effective for handling complex seasonal patterns^103^; (*iv*) TBATS (Trigonometric, Box-Cox transform, ARMA errors, Trend and Seasonal components), which are an extension of ETS that handle multiple seasonalities and complex seasonal patterns through a state space framework^105^; and (*v*) TSLM (Time Series Linear Model), which incorporates linear regression techniques to model the trend and seasonality directly, often with the inclusion of external regressors to capture exogenous influences on the time series.^103^

The comparison consisted in forecasting the dependent variable h steps ahead ($m_{E_{\text{avg}.\text{Entrp}}}^{t+h}$) and measuring its performance using several metrics in a cross-validation fashion. The time series cross-validation procedure used was a rolling forecasting origin procedure, in which models are trained on expanding windows over the time series, and their forecasts are evaluated against actual future values. Therefore, this experiment was repeated by increasing h by 1 until it was at most half the length of each experiment (for instance for Riet MZ04 experiment, and l = 8, a $m_{E_{\text{avg}.\text{Entrp}}}$ time series with 31 observations is obtained; thus, h = 1,...,15). The performance measures used to analyze the errors were the Mean Absolute Error (MAE) and the Root Mean Squared Error (RMSE).

The ARFIMA model obtained consistently the best results across the different forecasting horizons, as well as for each ind×ctx (Figure S1). Therefore, the ARFIMA model was used for carrying out the forecasting of the $m_{E_{avg.Entrp}}$ for all four sessions with the subjects. For each of these, the forecasting horizon was selected to be half of the length of the time series.
