# Supplementary material for: Generative vocal plasticity in chimpanzees
Source: iScience. 2025 Apr 8;28(5):112381. doi: 10.1016/j.isci.2025.112381 (PMC12049825; doi:10.1016/j.isci.2025.112381)
Supplement: Document S1. Figure S1 [file mmc1.pdf]

iScience, Volume *28*

Supplemental information

Generative vocal plasticity in chimpanzees

Adriano R. Lameira, Bruno Caneco, Arik Kershenbaum, Guillermo Santamaría-Bonfil, and Josep Call

Generative vocal plasticity in chimpanzees

Adriano R. Lameira[1*], Bruno Caneco[2a], Arik Kershenbaum[3,4a], Guillermo Santamaría-Bonfil[5a], Josep Call[6]

[1] ApeTank, Department of Psychology, University of Warwick, Coventry, UK
[2] DMP Statistical Solutions, St Andrews, UK
[3] Department of Zoology, University of Cambridge, Cambridge, UK
[4] Girton College, University of Cambridge, Cambridge, UK
[5] Data Portfolio Manager Department, BBVA Mexico, Mexico City, Mexico
[6] Department of Psychology and Neuroscience, University of St Andrews, UK
[a] Alphabetic co-author order
[*]Corresponding (lead) author: adriano.lameira@warwick.ac.uk

Supplemental figure

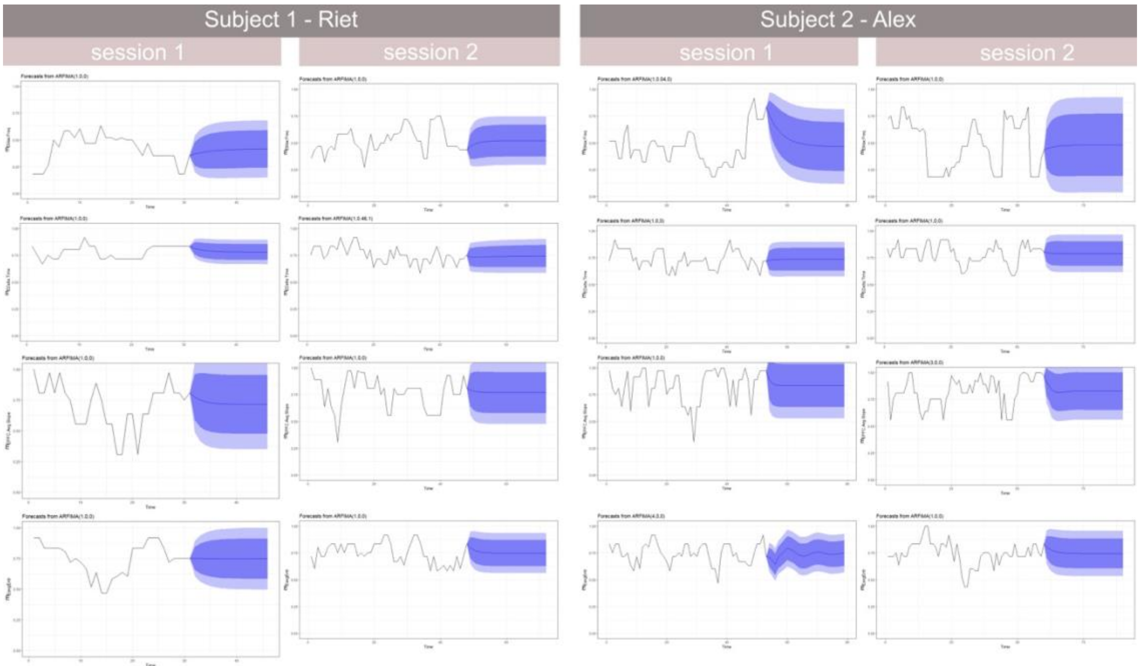

Fig. S1. Forecasting models of entropic emergence of atypical chimpanzee vowel-like voiced calls per session per individual. Blue bands represent 90% and 95% confidence intervals.
